# 

**Published:** 1883-10

**Authors:** 


Vol. IV. OCTOBER, 1883. No. 10.

THE

### Independent Practitioner:

A MONTHLY JOURNAL,

DEVOTED TO

MEDICINE, SURGERY, OBSTETRICS, DENTISTRY, PATHOLOGY AND POPULAR SCIENCE.

---

W. C. BARRETT, M. D., D. D. S.,

EDITOR.

COLUMBIA PUBLISHING COMPANY.

**$2.50 Per Annum in Advance. - - Single Copies, 25 Cents.**

Entered at the Post-Office, Buffalo, N. Y., as Second-Class matter.

